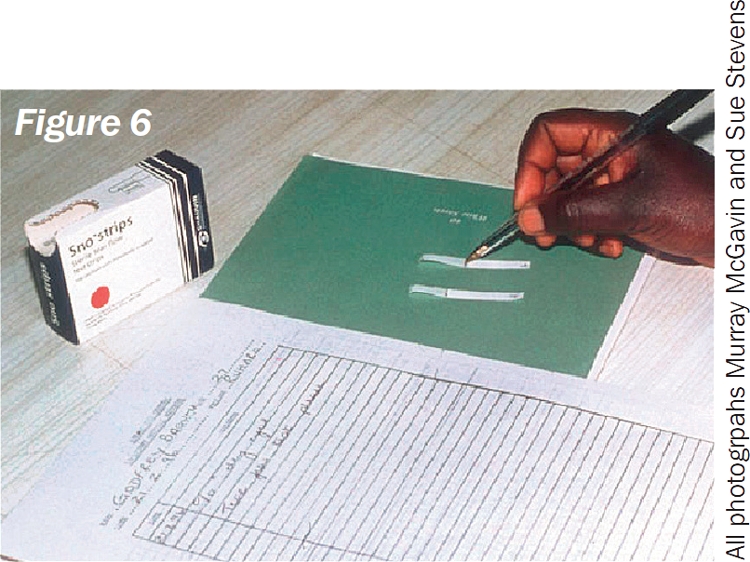# Schirmer's test

**Published:** 2011-12

**Authors:** Sue Stevens

**Affiliations:** Former Nurse Advisor, Community Eye Health Journal, International Centre for Eye Health, London School of Hygiene and Tropical Medicine, Keppel Street, London WC1E 7HT, UK.

**Figure:**
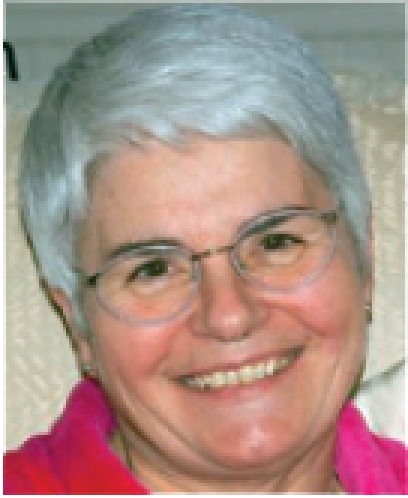
Sue Stevens

## Before performing any eye procedure

- Wash your hands (and afterwards too). Position the patient comfortably with head supported.
- Avoid distraction for yourself and the patient.
- Ensure good lighting.
- Always explain to the patient what you are going to do.

## Reasons for Schimer's test

To record measurement of tear secretion in patients with suspected ‘dry eyes’.

**Figure:**
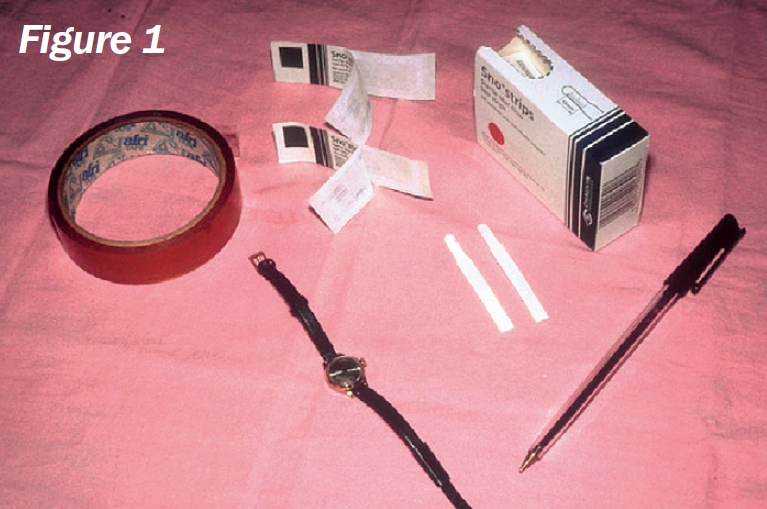


## You will need (Figure 1):

- Schirmer's test strips
- Watch or clock
- Clear adhesive tape
- Pen.

## Preparation

- Explain to the patient that although this procedure may be uncomfortable, it is not painful.

**Remember:** *Do not instil any anaesthetic drops or other eye medication before the test. This would give a false result.*

**Figure:**
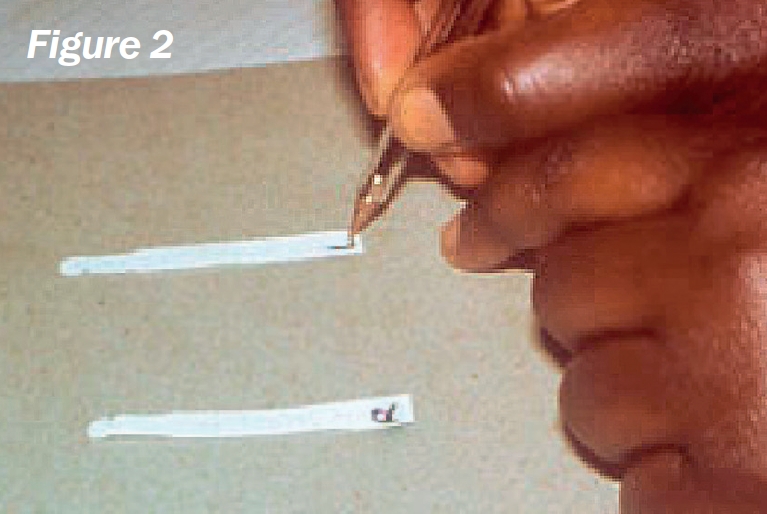


## Method

- Remove two strips from the sterile packet and label them ‘R’ (right) and ‘L’ (left) (Figure 2).
- Bend each strip, at the notch, to a 90 degree angle (Figure 3).
- Ask the patient to look up and, with an index finger, gently pull down the lower eyelid.
- Hook the bent end of the strip over the centre of the lower eyelid and allow it to ‘sit’ inside (Figure 4).
- Repeat the procedure for the other eye.
- Note the time (Figure 5).
- Ask the patient **not to squeeze**, but just to keep the eyes gently closed.
- After five minutes, ask the patient to open both eyes and look upwards.
- Carefully remove both strips.
- Using the package scale, measure the length of the moistened area on the strip, from the notch, and indicate this with a pen mark (Figure 6).
- Stick the strips into the patient's documentation and record the measurements below each strip, e.g., “10 mm in 5 minutes”. If the strips are completely moistened before five minutes, record appropriately, e.g., “30 mm in 3 minutes”.

**Figure:**
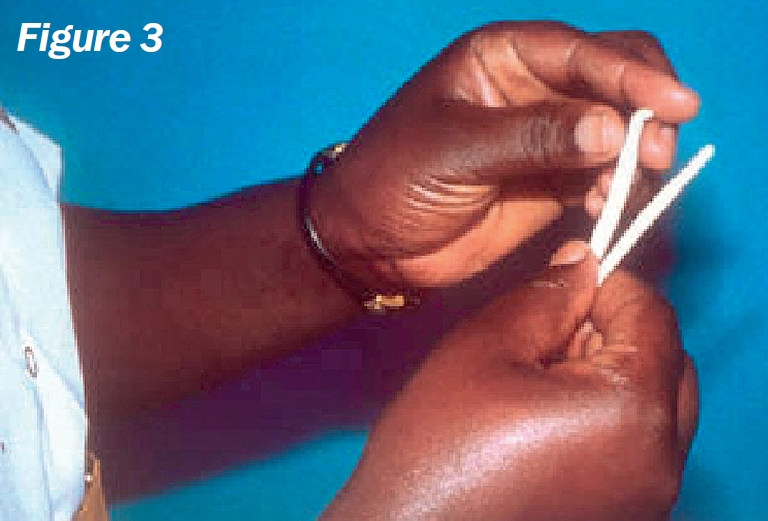


**Figure:**
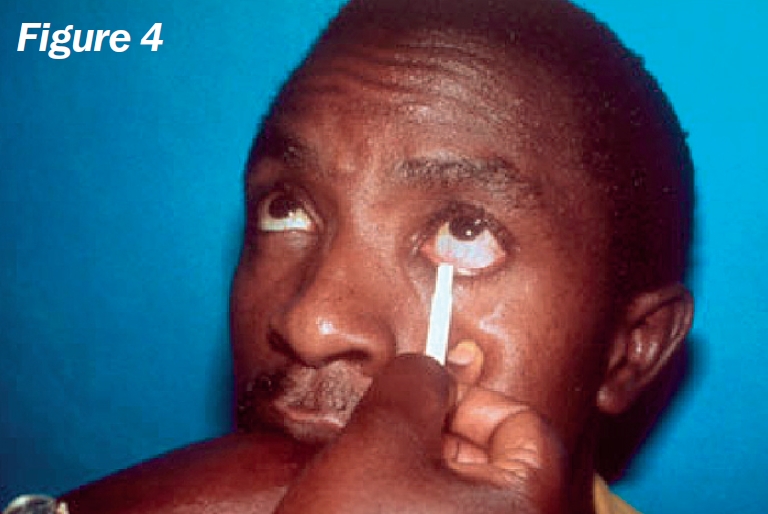


**Figure:**
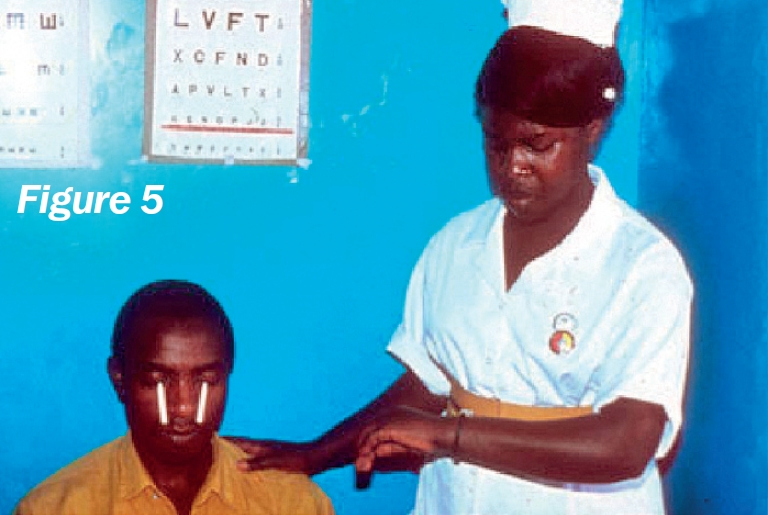


**Figure:**